# Sarcomatoid Intrahepatic Cholangiocarcinoma With Well‐Differentiated Squamous Cell Carcinoma Misdiagnosed as Liver Abscess: A Case Report

**DOI:** 10.1002/cnr2.70749

**Published:** 2026-11-08

**Authors:** Jing Yang, Ziyan Dong, Xinying Shi, Xin Luo, Jinfeng Cao

**Affiliations:** ^1^ School of Medical Imaging Shandong Medical and Pharmaceutical University Yantai China; ^2^ Department of Radiology Zibo Central Hospital Zibo China; ^3^ School of Medical Imaging Shandong Second Medical University Weifang China

**Keywords:** computed tomography, liver abscess, magnetic resonance imaging, sarcomatoid intrahepatic cholangiocarcinoma (sICC), well‐differentiated squamous cell carcinoma

## Abstract

**Background:**

Sarcomatoid intrahepatic cholangiocarcinoma (sICC) is a rare and highly aggressive variant of intrahepatic cholangiocarcinoma. Due to overlapping clinical and imaging features, it is frequently misdiagnosed as benign hepatic lesions, such as bacterial liver abscesses.

**Case:**

Herein, we report a case of a 59‐year‐old male patient who presented with fever and right upper abdominal pain. Laboratory tests revealed markedly elevated inflammatory markers. Ultrasound‐guided percutaneous drainage was performed, and *Hafnia alvei* was cultured from the purulent fluid. Combined with abscess‐like imaging manifestations and tumor necrosis with possible secondary infection, the case was preoperatively misdiagnosed as a bacterial liver abscess. The patient underwent a laparoscopic partial hepatectomy. Intraoperative frozen‐section pathology suggested a hepatic malignant tumor, and the final postoperative diagnosis confirmed sICC with well‐differentiated squamous cell carcinoma. Despite aggressive surgical intervention, the patient's condition rapidly deteriorated with imaging suggestive of tumor progression, and he died of multiple organ failure approximately 10 weeks after the initial surgery.

**Conclusion:**

This case underscores the diagnostic pitfall of sICC presenting as a liver abscess. Puncture or drainage of such necrotic masses should be performed with caution due to the risk of tumor cell dissemination. Early recognition, timely biopsy, and aggressive radical resection are crucial for improving the prognosis of this rare entity.

## 1 Introduction

Epithelial‐derived sarcomatoid tumors predominantly arise in the bladder, lung, and other organs, while they are extremely rare in the liver and biliary system. Sarcomatoid intrahepatic cholangiocarcinoma (sICC) accounts for approximately 4.5% of all intrahepatic cholangiocarcinomas and represents a rare, aggressive pathological variant [1]. Patients with sICC usually present with non‐specific clinical manifestations such as abdominal pain and fever, which lack distinctive diagnostic clues [2]. Radiologically, sICC commonly manifests as irregular hepatic masses with peripheral rim enhancement, exhibiting significant imaging overlap with benign and malignant hepatic lesions, particularly bacterial liver abscess and conventional intrahepatic cholangiocarcinoma. Such similarity leads to a high rate of preoperative misdiagnosis, and the definitive diagnosis relies strictly on histopathological and immunohistochemical examination [3]. Herein, we report a rare case of sICC initially misdiagnosed as a hepatic abscess. Combined with a literature review, we summarize the key causes of misdiagnosis and clarify the differential imaging and pathological features of this disease, aiming to improve clinical recognition and differential diagnosis of this rare malignant entity.

## 2 Case Presentation

A 59‐year‐old male patient was admitted to the Department of Hepatobiliary Surgery, Zibo Central Hospital, Shandong Province, in mid‐December 2024. He had intermittent fever for more than 2 weeks. Approximately 1 week before admission, he developed upper abdominal pain, and 2 days before admission, he had a recurrent fever with a maximum temperature of 37.9°C. The patient had no history of chronic diseases such as hepatitis or tuberculosis, but had a long‐term history of heavy smoking and alcohol consumption. Physical examination revealed tenderness in the right upper abdomen, and the liver was palpable below the costal margin with tenderness. Laboratory tests showed markedly elevated inflammatory indicators: the white blood cell count was 19.47 × 10^9^/L with a neutrophil proportion of 91.40%, and the C‐reactive protein level was 124.92 mg/L. Liver function parameters and serum tumor markers were within normal ranges. Ultrasound‐guided percutaneous puncture of the hepatic lesion yielded a small amount of bloody purulent fluid. Microbial culture of the pus confirmed *Hafnia alvei* , and antibiotic susceptibility testing indicated sensitivity to meropenem. After modification of the anti‐infective regimen, recurrent fever persisted without visible lesion liquefaction on imaging, indicating limited efficacy of antimicrobial treatment alone. Abdominal ultrasonography detected a mixed echo mass measuring approximately 8.2 × 7.1 cm within the hepatic parenchyma. Plain computed tomography (CT) scan revealed an inhomogeneous hypodense lesion in Hepatic Segment 4, with a maximum cross‐sectional size of 8.8 × 8.7 cm (Figure 1A). Dynamic contrast‐enhanced CT demonstrated peripheral wall enhancement of the mass with clearer demarcation compared with plain scanning (Figure 1B–D). Abdominal magnetic resonance imaging (MRI) showed heterogeneous slightly high signal intensity on fat‐suppressed T2‐weighted images (Figure 2A), high hyperintensity on diffusion‐weighted imaging (DWI) (Figure 2B), and decreased signal on the apparent diffusion coefficient (ADC) map (Figure 2C). The lesion presented hypointensity on T1‐weighted images (Figure 2D). Dynamic contrast‐enhanced MRI showed heterogeneous peripheral enhancement in the arterial phase and iso‐intensity relative to the surrounding hepatic parenchyma in the delayed phase (Figure 2E–I). Given the clinical manifestations of fever and abdominal pain, significantly elevated inflammatory biomarkers, and typical abscess‐like imaging features, the patient was preoperatively misdiagnosed with a bacterial liver abscess.

**FIGURE 1:**
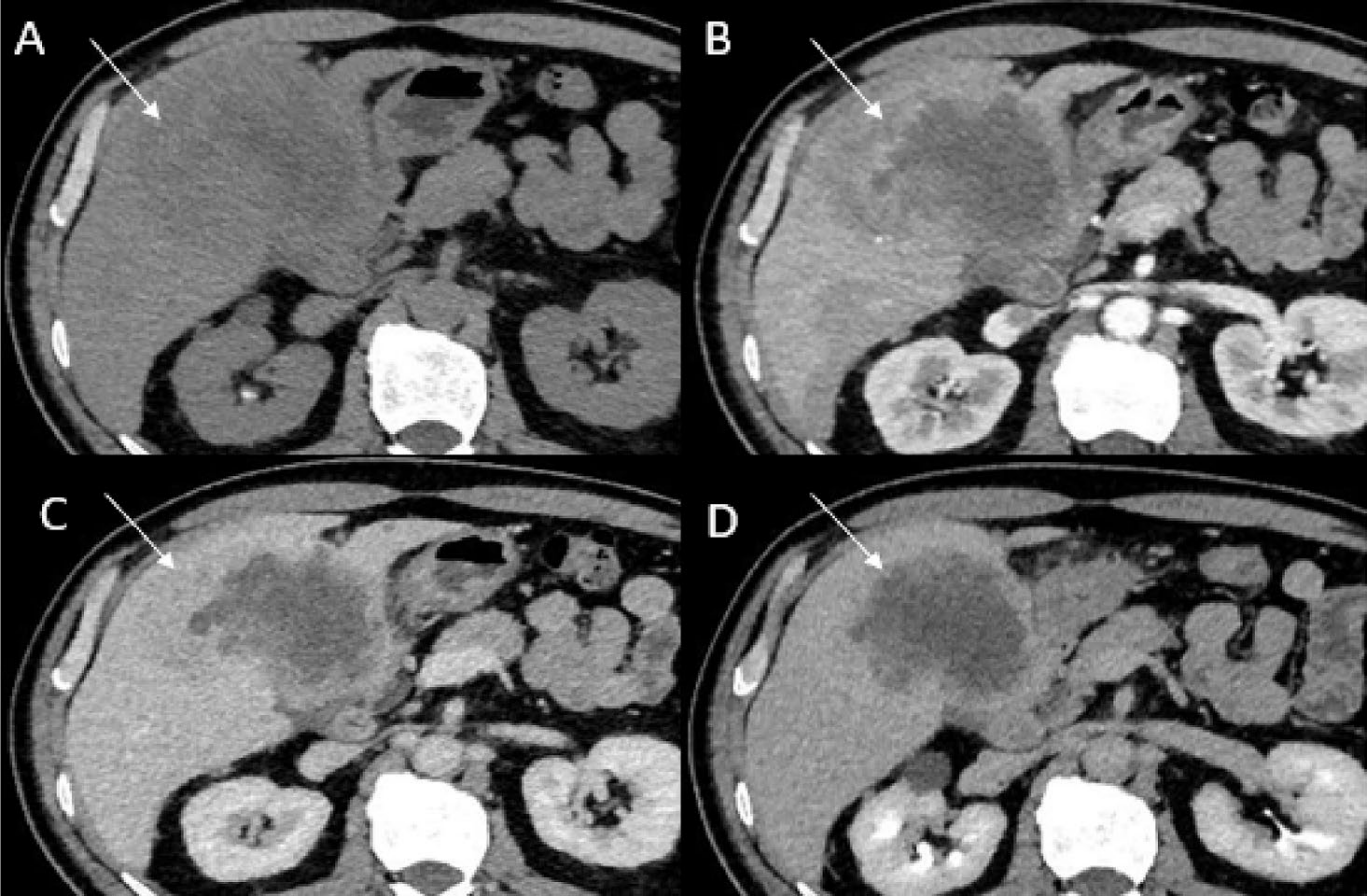
CT images from a 59‐year‐old male patient with a lesion located in Hepatic Segment 4, which was subsequently diagnosed as sarcomatoid intrahepatic cholangiocarcinoma (sICC). (A) Axial plain CT shows an inhomogeneous slightly hypodense lesion in Hepatic Segment 4. (B–D) Contrast‐enhanced CT demonstrates peripheral rim enhancement of the lesion, with a central non‐enhancing hypodense area consistent with liquefactive necrosis. Perihepatic fat stranding is observed.

**FIGURE 2:**
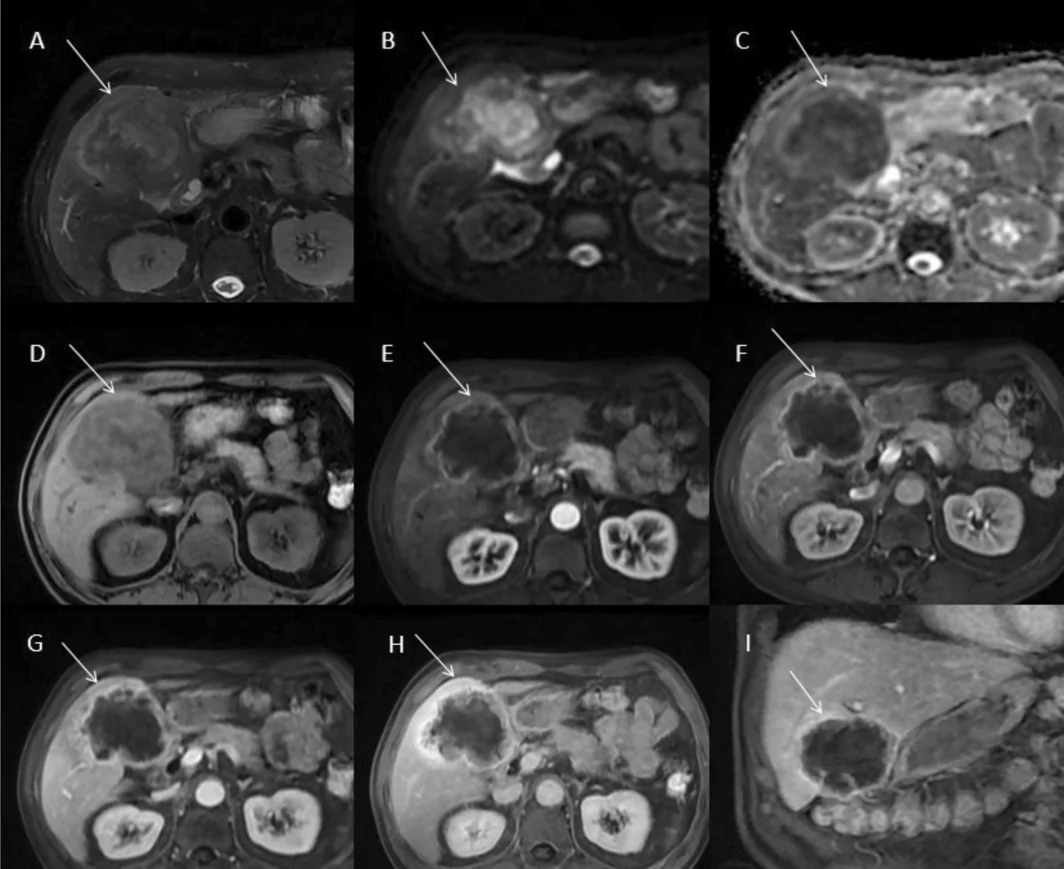
MRI images of the 59‐year‐old male patient. (A) Axial fat‐suppressed T2‐weighted image shows heterogeneous slightly high signal intensity within the lesion. (B) The lesion presents high signal intensity on DWI. (C) Low signal intensity of the lesion is noted on the ADC map. (D) T1‐weighted image demonstrates hypointensity of the lesion. (E–H) Axial contrast‐enhanced MRI reveals heterogeneous peripheral enhancement of the mass in the arterial phase, and the mass becomes iso‐intense relative to the surrounding hepatic parenchyma in the delayed phase. (I) Coronal contrast‐enhanced MRI shows enhancement of the lesion capsule.

The patient underwent laparoscopic partial hepatectomy plus intestinal adhesion lysis in late December, 2024. Intraoperative frozen‐section pathology confirmed a hepatic malignant tumor. Since the surgical strategy was preoperatively designed for a presumed liver abscess, the resection scope for radical tumor excision had not been fully evaluated. Limited by severe local inflammatory edema and operative conditions of the residual liver parenchyma, only local lesion resection was achieved in the initial operation, and extended radical resection with sufficient safe surgical margins could not be completed in a single stage. Given the malignant diagnosis on frozen section and favorable general condition without evidence of distant metastasis on systemic evaluation, reoperation was deemed feasible. Approximately 3 weeks after the initial surgery, extended radical hepatectomy was performed in mid‐January 2025. Postoperative pathology confirmed an intrahepatic cholangiocarcinoma with sarcomatoid differentiation and well‐differentiated squamous cell carcinoma. The tumor consisted of approximately 50% adenocarcinoma, 30% well‐differentiated squamous cell carcinoma, and 20% sarcomatoid spindle‐cell components. Pathological examination revealed tumor invasion of the gallbladder, duodenum, gastric wall, and full‐thickness colonic wall, with vascular tumor thrombi identified. Immunohistochemically, p53 was positive in 70% of tumor cells. Final pathological examination revealed metastatic carcinoma in the mesocolic lymph nodes (1/13), station 12 lymph nodes (1/1), station 13 lymph nodes (1/1, largest focus 4.5 × 2.8 × 0.8 cm), and the right hepatic artery lymph node (1/1). Metastatic carcinoma foci were identified in the pericecal extra‐serosal fat. Microscopic examination confirmed negative resection margins at the stomach, duodenum, colon, and ileum. However, the hepatic transection margin showed no normal liver tissue, indicating that the margin was extremely close to or involved by the tumor. Therefore, R0 resection was not achieved at the hepatic margin. According to the eighth edition AJCC staging system, tumor invasion of extrahepatic organs was defined as pT4. Metastasis to regional lymph nodes (Station 12 and the right hepatic artery node) was classified as pN1. Metastasis to the mesocolic lymph nodes, station 13 lymph nodes, and peritoneal deposits in the pericecal fat, which are outside the regional nodal basin, were classified as pM1. The final pathological stage was pT4N1M1 (Stage IV).

The patient recovered uneventfully and was discharged in early February 2025. Approximately 2 weeks after discharge, the patient was readmitted due to abdominal pain. During the physical examination upon admission, tumor metastasis was detected at the surgical incision, but this was only a clinical suspicion; no biopsy was performed. Postoperative CT follow‐up showed multiple hypodense lesions in the liver with progressive enlargement, enlarged abdominal and retroperitoneal lymph nodes, and progressive enlargement of right abdominal wall soft‐tissue lesions. New pulmonary opacities and nodules, bilateral pleural effusions, and a new right renal hypodense lesion were also observed. These findings were highly suggestive of tumor progression or distant dissemination; however, no biopsy was obtained due to rapid clinical deterioration, and inflammatory or benign causes could not be excluded. Given the patient's rapid postoperative progression and severely compromised performance status, active systemic therapy (including chemotherapy, targeted therapy, and immunotherapy) was not tolerated. Therefore, only palliative supportive care was provided. The patient died of multiple organ failure secondary to advanced malignancy (pT4N1M1). Postoperative complications included transient post‐hepatectomy liver failure (PHLF Grade A), recurrent fever, abdominal infection with *Enterococcus faecalis* , and hypoalbuminemia (Table 1).

**TABLE 1:** Timeline of the patient's main clinical events.

Time	Key clinical events
Mid‐December 2024	The patient presented with fever for more than 2 weeks and upper abdominal pain of 1‐week duration aggravated over 2 days, and was initially suspected of having a liver abscess.
Mid‐December 2024	Ultrasound‐guided percutaneous puncture and drainage of the hepatic lesion was performed. Pus culture was positive for *Hafnia alvei* .
Late December 2024	Laparoscopic partial hepatectomy combined with enterolysis was performed. Intraoperative frozen section suggested malignant hepatic tumor, and a sarcomatoid component could not be excluded.
Early January 2025	Combined pathological and immunohistochemical findings confirmed a diagnosis of intrahepatic cholangiocarcinoma with sarcomatoid differentiation and focal well‐differentiated squamous cell carcinoma.
Mid‐January 2025	Extended radical resection with combined multi‐visceral resection was performed. R0 resection was not achieved at the hepatic margin. Metastatic carcinoma was identified in several regional lymph nodes. The final pathological stage was pT4N1M1 based on the 8th edition AJCC staging system.
Early February 2025	The patient recovered well and was discharged from the hospital.
Mid‐February 2025	The patient was readmitted due to abdominal pain. A firm protruding mass at the surgical incision was clinically considered abdominal wall implantation metastasis. Repeated chest and abdominal CT revealed multiple abnormal manifestations including enlarged abdominal lymph nodes, intrahepatic lesions, and serous cavity effusion. Biopsy was not performed due to rapid disease progression. Extensive tumor progression was highly suspected, and palliative supportive treatment was administered.
Early March 2025	Clinical suspicion of tumor progression without autopsy confirmation; final cause of death was multiple organ failure.

## 3 Discussion

According to the 2019 WHO classification of digestive system tumors, sarcomatoid change in intrahepatic cholangiocarcinoma is a rare morphological variant characterized by spindle‐cell areas resembling spindle‐cell sarcoma, fibrosarcoma, or malignant fibrous histiocytoma; scattered foci such as squamous cell carcinoma may be observed in some cases [4].

Previous studies have confirmed that chronic hepatitis B infection and liver cirrhosis are established risk factors for sICC [5]. However, accumulating case reports have documented sICC patients without these classic predisposing conditions, which is consistent with the present case, as our patient had no history of hepatitis B or liver cirrhosis [6]. The patient in this study had a long‐term history of heavy smoking and alcohol consumption. Although epidemiological evidence has linked tobacco and alcohol exposure with conventional intrahepatic cholangiocarcinoma, no studies have verified them as specific risk factors for sICC [7, 8]. Therefore, sICC should not be excluded merely due to the absence of traditional hepatobiliary risk factors such as viral hepatitis and cirrhosis. The patient presented with right upper abdominal pain, fever, and elevated peripheral leukocytes, which mimicked typical infectious manifestations and largely contributed to the preoperative confusion with hepatic abscess. Imaging features, including a solitary large hepatic mass with ill‐defined borders, hypoattenuation on CT, heterogeneous hyperintensity on T2‐weighted MRI, central cystic necrosis, and peripheral or heterogeneous arterial enhancement, are suggestive but not pathognomonic for sICC. These overlapping radiological appearances necessitate careful differentiation from conventional cholangiocarcinoma and bacterial liver abscess [9]. Given the lack of specific clinical and imaging biomarkers for sICC, histopathological examination combined with immunohistochemical profiling remains the gold standard for definitive diagnosis [10].

Histopathologically [11], sICC is characterized by a typical biphasic histological structure, consisting of intermingled biliary adenocarcinoma and sarcomatoid spindle‐cell components, and squamous cell differentiation can be observed in a subset of cases. The dual immunohistochemical phenotype, with co‐expression of epithelial and mesenchymal markers, together with negative staining for HepPar‐1 and AFP, can effectively exclude other primary hepatic malignancies, particularly hepatocellular carcinoma. In the present case, microscopic examination confirmed the biphasic differentiation of the tumor. Residual biliary epithelial carcinoma nests and focal well‐differentiated squamous cell carcinoma with squamous cell nests and keratinization were identified. Abundant intersecting sarcomatoid spindle cells were diffusely distributed throughout the lesion. Significant cellular atypia was observed, with admixed and transitional areas among adenocarcinoma, squamous cell carcinoma, and sarcomatoid components (Figure 3A,B). Immunohistochemically, positive expression of epithelial markers including CK19 and pan‐keratin confirmed the biliary origin of the adenocarcinoma component (Figure 3C,D). Negative HepPar‐1 staining excluded a hepatocellular origin (Figure 3E), while diffuse vimentin positivity supported the presence of sarcomatoid spindle‐cell components (Figure 3F). The above pathological and immunohistochemical findings are consistent with previously reported features of sICC. The primary site was further confirmed to be intrahepatic based on the intraoperative finding of a tumor epicenter in liver segments IV and V, with direct invasion of adjacent organs. Immunohistochemically, negative staining for CK20, GPC‐3, and AFP additionally excluded colorectal, hepatocellular, and other gastrointestinal primaries. Although the patient had a heavy smoking history, preoperative chest CT and x‐ray showed no primary lung tumor. Endoscopy and PET‐CT were not performed because the preoperative diagnosis was a liver abscess; this is a limitation of the retrospective case.

**FIGURE 3:**
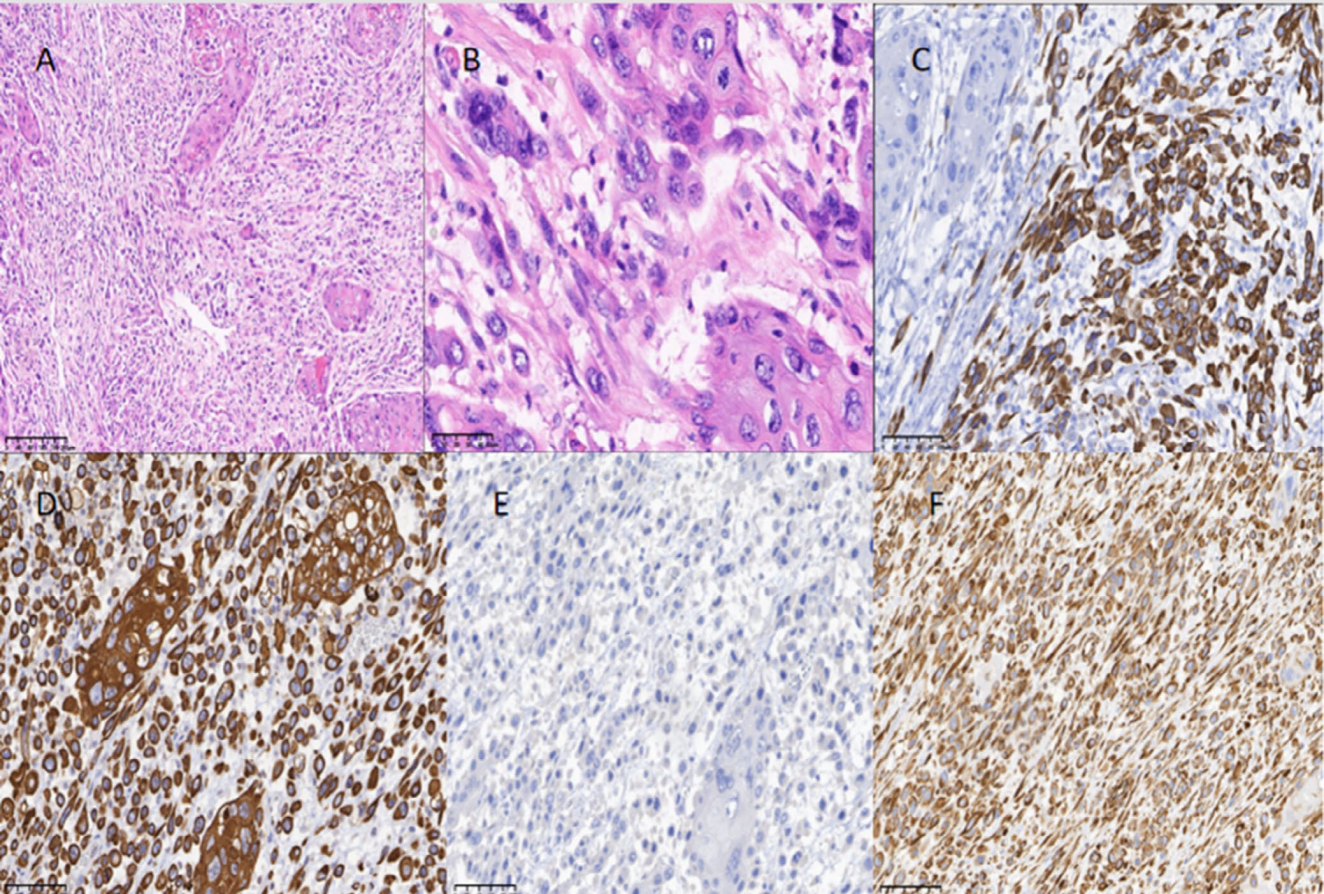
HE staining and immunohistochemical results of the tumor tissue from the second operation. (A) HE staining (×40, scale bar = 200 μm). The tumor contains adenocarcinoma nests, focal well‐differentiated squamous cell carcinoma, and abundant spindle‐shaped sarcomatoid tumor cells, with intermingled and transitional changes among these components. (B) HE staining (×400, scale bar = 50 μm). Marked cellular atypia is observed, showing multidirectional differentiation including adenocarcinoma, squamous, and sarcomatoid components. (C) CK19 (×200, scale bar = 100 μm): Positive expression in epithelial tumor components. (D) Pan‐cytokeratin (×200, scale bar = 100 μm): Positive expression in tumor components of epithelial origin. (E) HepPar‐1 (×200, scale bar = 100 μm): Negative staining in tumor cells, excluding hepatocellular carcinoma. (F) Vimentin (×200, scale bar = 100 μm): Diffuse positive staining in spindle‐shaped sarcomatoid tumor cells.

The tumor harbored three differentiated components, namely adenocarcinoma, sarcomatoid spindle‐cell elements, and well‐differentiated squamous cell carcinoma. The coexistence of all three components is uncommon in sICC. A summary of previously reported cases of sICC or intrahepatic cholangiocarcinoma with squamous differentiation is provided in Table 2. The intraoperative finding of purulent coating and bloody purulent drainage, together with the pathological evidence of extensive tumor necrosis and acute inflammatory infiltration, is consistent with a possible secondary infection of the necrotic tumor. However, because *Hafnia alvei* is an enteric organism and the tumors invaded the duodenum and full thickness of the colon, bacterial entry through the bowel wall is a plausible alternative. A coexisting simple abscess cannot be entirely excluded, although the solid nature of the tumors and the limited liquefaction on imaging argue against it. Postoperative imaging follow‐up revealed multiple hypodense lesions in the liver and surgical bed, enlarged abdominal and retroperitoneal lymph nodes, and multiple soft‐tissue density lesions in the right abdominal wall, which were highly suggestive of tumor recurrence and metastasis; however, no biopsy confirmation was obtained due to the patient's rapidly deteriorating clinical condition. This finding highlights the highly aggressive nature of this subtype and adds incremental evidence to the existing literature.

**TABLE 2:** Comparison of clinicopathological features between the present case and previously reported cases of intrahepatic cholangiocarcinoma with squamous differentiation.

Items	Present case	Liu et al. [6]	Sasaki et al. [12]	Nguyen et al. [13]
Age and Sex	59 years, male	76 years, male	79 years, male	72 years, male
Key immunohistochemical findings	CK19+, pan‐CK+, Vimentin+, HepPar‐1−; p53 positive (70%)	CK7+, CK19+, CK5/6+, p40+, Vimentin+; p53 mutation‐type	Keratin/EMA+ in carcinomatous foci; Vimentin+ in sarcomatoid regions	CK7+, CK19+, CK5/6+, p40+, Vimentin+, HepPar‐1−; INI1 retained
HBV/HCV status	No hepatitis B or C; long‐term heavy smoking and alcohol	Not mentioned	HBsAg, HBsAb, HBcAb all negative; HCV not mentioned	Hepatitis B and C serology negative
Clinical special feature	Mimicked bacterial liver abscess; pus culture positive for *Hafnia alvei*	No abscess‐like manifestation	No abscess‐like manifestation	No abscess‐like manifestation; no cirrhosis
Treatment strategy	Two‐stage hepatectomy combined with multi‐visceral resection; no subsequent systemic anti‐tumor therapy	Palliative hepatectomy followed by lenvatinib targeted therapy	No surgical resection; diagnosis confirmed by autopsy	Extended right hepatectomy with lymph node dissection; no effective adjuvant therapy
Tumor stage	pT4N1M1 (Stage IV)	Not reported	Not reported (diagnosed at autopsy)	Not reported
Clinical outcome	Died of multiple‐organ‐failure approximately 10 weeks after surgery	Died of cardiopulmonary failure 8 months after surgery	Died of respiratory failure	Died of liver failure 3 months after surgery

Combined with previous literature [3], the key reasons for misdiagnosis of sICC as liver abscess in the present case are summarized as follows. (1) Overlapping clinical manifestations: sICC with central tumor necrosis and inflammatory reaction can induce systemic infectious symptoms, including fever and leukocytosis, which are identical to the clinical presentations of bacterial liver abscess. Pus culture from percutaneous hepatic drainage yielded *Hafnia alvei* in this case, which is consistent with a possible secondary infection within the necrotic tumors lesion. The patient's fever and elevated inflammatory markers resulted from the combined effects of inflammatory mediators released by tumor necrosis and local bacterial infection in the lesion, and the positive microbiological findings further confused the clinical judgment. (2) Similar imaging features: sICC commonly presents as round‐like hypodense lesions on CT and prolonged T2 signal on MRI, with peripheral rim enhancement on contrast‐enhanced scanning. Such manifestations highly resemble those of necrotic bacterial liver abscesses, lacking specific differential imaging signs. Accordingly, it is difficult to distinguish sICC from hepatic abscess merely based on imaging findings. This case emphasizes that for abscess‐like necrotic hepatic masses, a positive pus culture alone is insufficient to confirm a simple bacterial liver abscess. Clinically, such lesions should be included in close follow‐up cohorts rather than being empirically diagnosed as benign abscesses. If standardized anti‐infective therapy achieves only transient improvement in infectious symptoms such as fever and leukocytosis, or if follow‐up imaging shows no obvious lesion absorption, or even progressive changes including increased solid components or honeycomb‐like necrosis, a malignant tumor with progressive tumor necrosis rather than simple inflammatory abscess should be highly suspected.

Radical surgical resection is the primary curative treatment option for sICC. However, due to its highly invasive biological behavior and propensity for early distant metastasis, most patients are no longer eligible for radical surgery at the time of definitive diagnosis [14]. sICC is associated with an extremely poor overall prognosis. Previous studies have demonstrated that the median survival of patients who undergo radical resection is approximately 11 months, while untreated patients have a median survival of only 3 months [15]. Moreover, adjuvant therapies including radiotherapy, chemotherapy, and targeted therapy yield limited survival benefits, and there is currently no standardized postoperative adjuvant regimen that can significantly improve patient prognosis [16]. Notably, several recent studies have reported promising outcomes of programmed death‐1 (PD‐1) inhibitor combined with anti‐angiogenic therapy for recurrent and metastatic sICC. In these cases, patients achieved partial response with a survival time of up to 12 months, providing a novel therapeutic strategy for this refractory malignancy [17].

The present study has several limitations. First, this is a single‐case report. Given the extreme rarity of sICC combined with well‐differentiated squamous cell carcinoma, the findings summarized from a single case cannot be fully generalized to all patients with this rare tumor subtype. Second, the patient's smoking and alcohol history was self‐reported without objective documentary evidence, which may introduce recall bias. Third, postoperative imaging suggested extensive tumor recurrence and metastasis, but no biopsy confirmation was obtained due to the patient's rapidly deteriorating clinical condition. Therefore, the long‐term evolutionary characteristics of this rare tumor could not be further analyzed.

In conclusion, the diagnosis of sICC relies on comprehensive integration of clinical, imaging, and pathological features. Pathological examination, particularly immunohistochemical staining, plays an irreplaceable role in definitive diagnosis and differential diagnosis. It should be noted that puncture or drainage of necrotic tumors may carry a risk of cancer cell dissemination. In biliary tract tumors and other solid tumors, the reported incidence of catheter tract implantation metastasis after percutaneous transhepatic biliary drainage (PTBD) is approximately 6% [18]. More recent studies have confirmed a significantly higher incidence of seeding metastasis in the PTBD group compared with the endoscopic drainage group, ranging from 22.0% to 26.7% [19, 20]. Needle tract seeding after percutaneous biopsy has been reported in approximately 1.6%–2.6% of cases [21], and this risk may be higher in necrotic tumors. These data suggest that the diagnostic benefits and procedure‐related risks should be carefully weighed in the management of necrotic tumors. Future studies should focus on developing more accurate non‐invasive diagnostic methods and safer therapeutic strategies to improve the prognosis of patients with this rare and aggressive malignancy.

## Author Contributions

**Jing Yang:** writing – original draft, investigation, writing – review and editing. **Ziyan Dong:** investigation. **Xinying Shi:** investigation. **Xin Luo:** writing – review and editing. **Jinfeng Cao:** writing – review and editing, investigation.

## Funding

The authors have nothing to report.

## Ethics Statement

This retrospective case report was conducted in accordance with the Declaration of Helsinki. The need for ethics committee approval was waived by the Ethics Committee of Zibo Central Hospital due to the retrospective nature of the study and the use of anonymized clinical data.

## Consent

Written informed consent for publication of this case report and relevant images was obtained from the patient's family. All identifiable patient information has been removed from the manuscript and figures to safeguard patient privacy.

## Conflicts of Interest

The authors declare no conflicts of interest.

## Data Availability

The data that support the findings of this study are available on request from the corresponding author. The data are not publicly available due to privacy or ethical restrictions.
